# A new classification method using array Comparative Genome Hybridization data, based on the concept of Limited Jumping Emerging Patterns

**DOI:** 10.1186/1471-2105-10-S1-S64

**Published:** 2009-01-30

**Authors:** Tomasz Gambin, Krzysztof Walczak

**Affiliations:** 1 Faculty of Electronics and Information Technology of Warsaw University of Technology, Institute of Computer Science, Nowowiejska 15/19, Warsaw, 00-665, Poland

## Abstract

**Background:**

Classification using aCGH data is an important and insufficiently investigated problem in bioinformatics. In this paper we propose a new classification method of DNA copy number data based on the concept of limited Jumping Emerging Patterns. We present the comparison of our limJEPClassifier to SVM which is considered the most successful classifier in the case of high-throughput data.

**Results:**

Our results revealed that the classification performance using limJEPClassifier is significantly higher than other methods. Furthermore, we show that application of the limited JEP's can significantly improve classification, when strongly unbalanced data are given.

**Conclusion:**

Nowadays, aCGH has become a very important tool, used in research of cancer or genomic disorders. Therefore, improving classification of aCGH data can have a great impact on many medical issues such as the process of diagnosis and finding disease-related genes. The performed experiment shows that the application of Jumping Emerging Patterns can be effective in the classification of high-dimensional data, including these from aCGH experiments.

## Background

### Introduction

Array-based Comparative Genomic Hybridization (aCGH) is a powerful technique used to detect DNA copy number variations (CNV) across the genome. One of the most important aims of this technique is diagnosis, which can be achieved with help of classification of aCGH data.

One of the most important problems with the classification of aCGH data is dealing with a great number of attributes, which often exceed the number of given samples. In a typical experiment one can deal with dozens of samples, while microarray may consist of millions of spots. It is a real challenge to select from the huge amount of data the most interesting features, while most of them are not related to the given classification problem.

In reference to this issue, reduction of the dimensionality of data by applying feature elimination algorithms has been proposed in the previous works [1,2]. One of the most interesting approaches, which is based on interval merging, has been presented in [1]. Unfortunately, the solution described by the authors contains some fallacies, which result from an improper mix of training and test data in their algorithm. In the Methods section, we revise interval merging approach and we propose a new corrected version.

The other problem of processing aCGH data is the unbalance in class distribution. For instance, it is quite common that the number of samples from one class exceeds by several times the number of samples from the other class. This makes classification much harder, and most of algorithms assume an uniform class distribution.

Moreover, very often, one specified class is much more important than others. Let's suppose, we want to distinguish two groups of individuals: healthy and unhealthy. We will concentrate more on unhealthy class. The mistake made by classifying the unhealthy as healthy (False Negative) is more costly than an opposite mistake (False Positive). It implies the necessity of being careful in analysing and comparing the quality of classification models.

On the other hand, it is common in practice, that researchers present the results of classification performance by showing only the accuracy [2,1,3]. Obviously, it is an insufficient measure, when unbalanced data are given. This problem is discussed more deeply in the Results and Discussion section. Obviously, the wrong assumption about evaluation measures leads to incorrect conclusions about classifiers performance. In this paper, we show that the classification methods considered most successful for high-dimensional data extremely decrease their performance dramatically when the samples distribution is out of balance. Therefore we decided to develop a new classification method, which is based on the concept of Jumping Emerging Patterns (JEP). The difficulties of algorithmic design, such as computational complexity and limitations, are explained in detail in the Methods section.

Thanks to very high discriminative power of JEP's, our limJEPClassifier could be more appropriate in classifying aCGH data than other methods. In order to compare our new algorithm to SVM [4], we prepare an experimental pipeline, precisely described in the section Methods. Finally, in the Results and Discussion section, we present the results of this comparison.

In the following section, we present details of aCGH technology.

### aCGH technology

Comparative Genome Hybridization (CGH) is a technique which allows for detection of segmental DNA copy number changes (CNC's) [5-7]. Recently, CGH has been widely used in many medical applications. In particular, it helps in the diagnosis of cancer [8] or genomic disorders [9], improves our knowledge about genes responsible for diseases and advances studies on personal genomic differences between humans [10].

**Figure 1:**
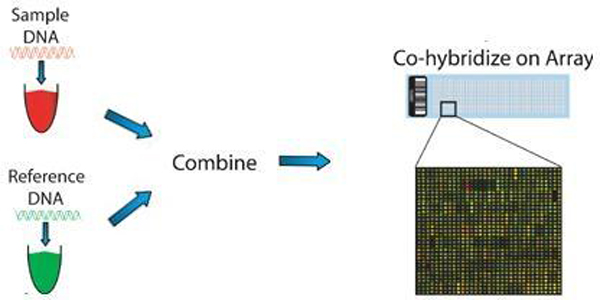
**Workflow of aCGH experiment**. Representation of aCGH technique [30].

In array-based CGH (aCGH) experiment, two differentially labeled samples are co-hybridized to targets, where the copy number between the two samples is reflected by their signal intensity ratios. In Figure 1 we present essential steps in this technique.

Through the years, the technology was upgraded to obtain a higher resolution by reducing length of DNA targets (probes). Nowadays, some aCGH platforms (e.g. oligonucleotide aCGH) support arrays with more than 1,000,000 probes.

Another kind of technique is SNP arrays (single nucleotide polymorphism), which differs from aCGH technology by its higher resolution. It allows to detect very small DNA changes.

Because of the huge amount of data, which are processed in aCGH experiments and some imperfections in technology, it becomes necessary to use the wide range of informatics and statistical tools at each stage of the analysis.

The first algorithmic challenge appears already in the preparation step. The issue is how to choose suitable probes, which will not cross-hybridize with other targets in the array. In this context several approaches have been proposed [11,12].

The next problem is related to the post-processing of signal data obtained from microarray. Because of many circumstances, which may affect results, it is essential to perform a normalization procedure, described in [13].

To obtain more clear results, smoothing, segmentation and aberration calling are usually used [2,14-16]. Nowadays, it is the most investigated area of aCGH analysis.

As we mentioned above, aCGH data is used in a diagnostic process. At this point a classification method has to be applied. In several papers, it has been suggested that the most accurate is a Support Vector Machine (SVM) [4]. In the next sections, we will evaluate this statement, by comparing SVM to our new classifier based on Jumping Emerging Patterns.

### Related works

In recent works, we have found a lot of information about processing aCGH data, such as smoothing and clustering [2,14-16]. However, there are many fewer papers, which deal with a classification problem.

In [17] and [18] the classifications of combined data (aCGH and gene expression or steorological data) are considered.

Another work [3] investigates the tumor classification based on DNA copy number aberration determined using SNP arrays. In that paper three classification methods: Naive Bayes, K-nn, and SVM have been tested with a varying number of features. Although the K-nn achieved the best leave-one-out cross-validation accuracy, the performances of other methods were comparable. The second conclusion of this work was that the best performance of classifiers was achieved when 5–30 features were selected. This fact reveals the importance of feature reduction algorithm in the classification of high-dimensional data. A new feature elimination methods for cancer classification using aCGH data have been proposed in the article [1]. Authors introduce a feature reduction algorithm based on an Interval Tree. In order to prove its efficiency, they compare the performance of a SVM classifier on two types of data: 1) raw log2ratio data; 2) data processed by their feature reduction algorithm. The results show that their approach led to a significantly better classification. Although the feature reduction procedure described by authors seems to be very useful, we have to point out some fallacies that we have found in their argumentation.

The first problem concerns the improper use of cross-validation. The cross-validation assumes that at the beginning of each run, data should be split into training and test data sets, and then all the operations before testing step should be done on both sets separately. However, in the system presented in [1], the feature reduction based on Interval Tree was performed on training and testing data together. In other words, the knowledge about test samples is utilized indirectly to train the model, which is clearly improper. The second major drawback of experimental design is that the authors used only one measure to evaluate a classification performance – the accuracy. We assume that it is insufficient and does not show a real classification performance, since one of the tested data sets (TP53) [19], is strongly unbalanced. In our paper, we investigate the same TP53 data set with revised experimental pipeline and more adequate evaluation measures. Beside testing the SVM, we also check our new method – limJEPclassifier.

## Methods

### Jumping Emerging Patterns

The classifiers based on Emerging Patterns (EP) or Jumping Emerging Patterns (JEP), have been considered one of the most successful classification systems [20]. However, it has hardly ever been tested with high-throughput data, because of the high computational complexity of EP. In this paper, we show how to overcome this issue by using "Limited JEP's".

First of all we present the definition of Jumping Emerging Patterns.

**Definition 1**. Given two data sets D1, D2 we define a Jumping Emerging Pattern from *D*1 to *D*2 as an itemset *X *(an attribute value pairs), for which *supp*_*D*1_(*X*) ≠ 0 and *supp*_*D*2_(*X*) = 0.

We will denote each JEP from class P to class N by attribute-value pairs.

**Definition 2**. The minimal JEP from P to N, which contains pattern (itemset) *X*, is such a JEP for which, does not exist a JEP, which contains pattern *Y *and *Y *⊂ *X*. In other words, JEP is minimal when all the patterns contained in its itemset do not form any other JEP.

**Example 1**. Consider a decision table – Table 1. We have given two data sets – classes P and N. By the definition patterns: {(a2, 1)}; {(a3, 1), (a4, 1)}; {(a2, 1), (a3, 1), (a4, 1)} are examples of JEP's, from class P to N. However the JEP {(a2, 1), (a3, 1), (a4, 1)} is not minimal, because it contains itemsets {(a2, 1)}, which form other JEP.

**Table 1:** Example of decision table

nr	a1	a2	a3	a4	Class
1	0	0	0	0	P
2	1	0	0	0	P
3	0	1	1	0	P
4	0	1	1	1	P
5	1	0	0	0	N
6	1	0	0	0	N
7	0	0	1	0	N
8	0	0	0	1	N

In this paper we consider only minimal JEP's.

### Limited JEP's

**Definition 3**. Limited JEP's at the level K are a set of minimal JEP's, for which the number of attributes in each itemset equals K. We denote limited JEP's at level K by *limJEP*_*K*_.

**Example 2**. According to the table 1, there are:

• one *limJEP*_1 _from P to N - {(a2, 1)};

• one *limJEP*_2 _from P to N - {(a3, 1), (a4, 1)} and three *limJEP*_2 _from N to P - {(a3, 0), (a4, 1)}, {(a2, 0), (a4, 1)}, {(a2, 0), (a3, 1)};

• no *limJEP*_3 _and *limJEP*_4_.

The major problem with classifiers based on JEP's is their high complexity, which increases rapidly with the number of attributes. In [21] the authors show that the emerging pattern problem is MAX-SNP hard. In order to overcome this issue several algorithms were developed, which significantly reduce the computation time: JEPproducer [22], CP-tree [23], FP-tree [24,25], classifier based on local projected JEP [26]. However, even with those methods it is still not possible to search all JEP's when the number of features exceeds few dozens.

In this paper, we claim, that in the case of high-throughput data, we can build a classifier based on limited JEP's for lower *limJEP *levels only, instead of computing all the patterns. In the following sections, we will show that this solution leads us to the construction of an efficient, and successful classifier. Below, we present how the discriminative power of JEP's may vary among levels of limited JEP's.

### REAL and UNREAL JEP's

We introduce the concept of REAL and UNREAL JEP's to illustrate the quality of patterns at different *limJEP *levels.

**Definition 4**. Let *U *denote a decision table of itemsets, where two classes (*P *and *N*) are given. From table *U *we select a subset of samples denoted by *A*. We say that the pattern *p *is a REAL JEP in decision table *A *⇔ if the *p *is still a JEP in table *U*. Otherwise *p *is UNREAL. We will denote all JEP's (REAL ∪ UNREAL) as ALL.

**Example 3**. Consider Table 1 as *U*. Suppose we select from *U *rows 3,4,5 and 6. We denote the new table by *U'*. Consider two patterns: *p*1 = {(*a*1, 0)} and *p*2 = {(*a*2, 1)}. Both patterns are JEP's in *U'*, however only *p*2 is still a JEP in *U*. In reference to definition 4, *p*1 is UNREAL JEP, and *p*2 is *REAL *JEP in *U'*.

In our study ratios of REAL to ALL JEP's are used, to measure the quality of *limJEP's *at the given *limJEP *level.

**Proposition 1**. For a given decision table *U *with the number of rows |*U|*, let *U' *be a training data set derived from *U*. Similarly, we denote *U" *as a set of rows selected from *U'*. We assume, that |*U'*| - |*U"*| ≪ |*U"*|. It means that a great majority of rows was selected. Then, if we compute JEP's for tables *U*, *U' *and *U"*, we can easily determine the number of REAL JEP's in *U" *with respect to *U' *and the number of REAL JEP's in *U' *in respect to *U *for each of the *limJEP *level. We put it that for a given *limJEP *level the ratio REAL/ALL JEP's is almost constant among tables *U" *and *U"*.

The proposition 1 allows us to infer from the training data about the quality of JEP's at a given *limJEP *level without a knowledge about test samples. Note, that in order to obtain the quality of investigated decision table *U*, we need to use only the training data (*U'*) and the table *U" *which is derived as a subset of *U'*.

We tried to confirm proposition 1 by testing TP-53 data set, described in the next section. Because of the high computational complexity, we used a decision table with the number of features reduced to 50. Data set was processed 10 times, as follows:

1. From original data set (*U*) we select randomly 80% of rows as training data (*U'*).

2. From *U' *we select 90% of rows, and denote it by *U"*.

3. We compute all JEP's at *limJEP *levels 1, 2 and 3 for tables *U*, *U' *and *U"*.

4. We calculate and compare REAL/ALL JEP's ratios in *U' *and *U" *at each level.

In Table 2 we present three samples of REAL/ALL ratios at each *limJEP *level and mean ratios from whole experiment (10 runs).

**Table 2:** REAL/ALL ratios Comparison of REAL/ALL ratios at different *limJEP *levels between tables *U' *and *U'*.

	REAL/ALL JEP's		
	limJEP1	limJEP2	limJEP3

run 1-*U'*	0.97	0.96	0.65
run 1-*U"*	1	0.97	0.67

run 2-*U'*	1	0.98	0.63
run 2-*U"*	1	0.96	0.68

run 3-*U'*	0.97	0.96	0.65
run 3-*U"*	1	0.97	0.67

mean-*U'*	0.97	0.9	0.53
mean-*U"*	0.98	0.85	0.5

As we can see, REAL/ALL ratios behave similarly for *U' *and *U"*. For a fixed *limJEP *level the differences in ratio between *U' *and *U" *at each run are relatively low. What is more, all the rows preserve the same, descending order of ratios. The best score is observed for *limJEP*_1_, next for *limJEP*_2 _and *limJEP*_3_. We have mentioned above that proposition 1 gives us the possibility to predict a quality of JEP's at given *limJEP *level. It seems reasonable, that in a similar way, we can investigate other features of our data set, such as the distribution of REAL JEP's in classes. This could be valuable information, especially when we build a classifier, which works with unbalanced data.

### Hierarchical strategy of classification

The fundamental application of JEP's is a classification. During the training step a classification model is built, which means that JEP's contained in training data are computed. In order to classify new data, the test sample is compared with all JEP's from the model. If the given JEP matches the sample, the algorithm increments the support for a class, which is associated with this JEP. Finally, the class with the greatest support is selected as a result.

**Example 4**. Suppose that the Table 1 is the given training data set. In this case, the derived model will contains two minimal JEP's from class P to N: {(a2, 1)}, {(a3, 1), (a4, 1)}; and three from N to P: {(a2, 0), (a3, 1)}, {(a2, 0), (a4, 1)}, {(a3, 0), (a4, 1)}. Consider the test instance {1, 1, 1, 1}. The classifier searches for all JEP's in the model which match this instance. In particular example there are two such JEP's from class P to N and no JEP's from class N to P. Based on this knowledge, the algorithm can easily decide that test sample should be classified as class P.

The strategies of classification with JEP's usually take into consideration the whole population of found JEP's [20]. Several algorithms filter or weight JEP's in reference to their support in the training data set [23].

Unfortunately, these approaches did not prove useful in the case of aCGH data. The accuracy of classification performed by traditional JEP techniques was revealed to be lower than the accuracy of other classifiers such as SVM. What is more, these methods are computational intensive when we deal with high-throughput data.

These facts motivated us to look for a more accurate strategy of JEP's classification. In this paper, we propose a new algorithm of classification with Jumping Emerging Patterns named limJEPClassifier which is based on the knowledge of variability of discrimination power at different *limJEP *levels. To the best of our knowledge, up to now there was no strategy that refers to *limJEP *levels. The steps of algorithm are presented below.

For a given model, computed sets of *limJEP's *at levels 1, 2,..., *m*, where *m *≤ number of attributes, limJEPClassifier processes a test sample as follows:

1. Sorts levels of limJEP's in reference to REAL/ALL ratio in the way explained in proposition 1.

2. Selects the best level and tries to classify the sample with JEP's from this level.

3. If success (support of one class dominates another one), returns the result; or else removes the last-considered level and goes back to point 2.

4. If no class is selected, chooses the most frequent class or randomizes it from appropriate distribution.

It is important that the REAL/ALL ratio is derived from the table *U"*, which is constructed based on the samples selected from a training data.

The other thing which is worth noticing, is that in our implementation of presented algorithm, we mainly use only the first two *limJEP *levels: *limJEP*_1 _and *limJEP*_2_. We found out that applying more levels does not improve the performance of classifier.

In the result section, we will show the advantages of our approach over other classification methods.

### Classification pipeline

**Figure 2:**
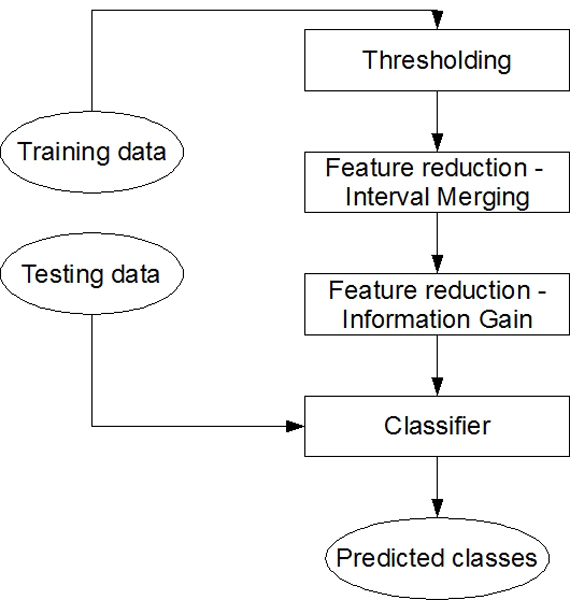
**Experimental pipeline**. Experimental pipeline for testing classifiers.

In order to test various classification models, we have developed a pipeline which includes preprocessing, model building and result analysis steps. The whole pipeline is presented in Figure 2. Below, we describe each step in detail.

#### Thresholding

In this step, we apply thresholding for all the data in order to determine normal, gain and loss regions. This procedure is done with the two values which indicate positive and negative thresholds, as follows:

• If the value of spot is above the positive threshold, we mark it as a gain.

• If the value of spot is below the negative threshold, we mark it as a loss.

• In other cases, spot is marked as normal.

For each spot, we assign new values: 0 for normals, 1 for gains and -1 for losses.

#### Sampling training data

In order to perform cross-validation we sample training data from the table achieved in the previous step. The following operations are performed only on training data until testing stage.

#### Feature reduction

Feature elimination is divided into the following two steps:

• Applying feature reduction algorithm based on interval tree (mentioned in the previous section).

• Selecting the most valuable features obtained by Information Gain approach.

Below we present both algorithms in detail.

#### Merging intervals

The core idea of this algorithm is to compress the data by merging segments (continuous sequences of spots with equal values). Arisen intervals can be used as new features. In the procedure all continuous sets of columns with the same values in each row are retrieved and transformed into one single column.

It is clear that the derived-in-this-way attributes, contain more statistical information about distribution in classes than the previous set with separated columns. This statement was confirmed in the paper [1], in which it was shown that the usage of this procedure significantly improved the accuracy of classification. It is worth noticing that the presented algorithm was applied only for training data, unlike in article [1]. We claim that such an attitude is more proper in relation to classification problem, because we do not use any test data during the training step.

However, this approach involves some complicating circumstances. Note that each sample from the test data has to be adjusted into a structure of a table derived from the training data set. In order to do that, it is required to transform a test sample by combining the same sets of columns, which were merged in the training data table. The main problem is how to assign proper values for merged attributes. In the case when all the values are equal, it is trivial and we assign this value. Otherwise, we select heuristically the value which appears most frequently in the given interval. We claim that this problem should be more deeply investigated in future research.

#### Information gain

Although applying the merging interval algorithm significantly reduces the number of attributes, there are still a great number of columns left. We decided to use an Information Gain approach [27] to weight the importance of each feature and sort them in a descending order. The algorithm measures the number of bits of information obtained for category prediction by knowing the presence or absence of a feature. The Information Gain of feature F is defined as:

$$\begin{array}{r} IG(f)=-\sum_{i=1}^{m}P(y_{i})\log P(y_{i})\\ +\sum_{v\in V}\sum_{i=1}^{m}P(f=v)P(y_{i}|f=v)\log P(y_{i}|f=v), \end{array}$$

where *y*_*i *_: *i *= 1...*m *are the set of categories and *V *set of possible values of *f*. At the end, the fixed number of top-ranked features are selected in order to form a final decision table.

#### Classification

When we obtain a final decision table, we train on it two classification models SVM (Support Vector Machine) [4] and limJEPClassifier.

The first classifier, SVM, was selected because it is commonly used in the case of high dimensional data. What is more, there can be found many applications of SVM to microarray gene-expression data, which are quite similar to aCGH data.

The core idea of SVM is to construct a separating hyperplane in the space of n-dimensional data between two sets of vectors (samples from two classes). The hyperplane should maximize the margin, defined as a sum of distances from hyperplane to the closest positive and negative samples [1].

The second classification method is our new approach – limJEPClassifier which was described in the previous section.

### Data source

In our study we used a commonly investigated data set – TP 53, published by [19]. The data are freely available at [28]. The aCGH data come from BAC arrays hybridized with *oral squamous cell carcinomas *(SCCs). The data set contains 14 TP53 mutant samples (unhealthy subjects) and 61 wildtype samples (healthy subjects). Each sample in a data set is featured by 1975 clones (log2ratio values).

## Results and discussion

### Evaluation measures

A typical problem with aCGH data is an unbalanced class distribution. In the case of the considered TP53 data set, the proportion between classes is 14:61. Moreover, the weight of each class is different. The rare class of sick subjects (14 samples) is more significant than the large set of samples of a control group. Traditionally, the rare and more important class is marked as "positive", while prevalent class is called "negative".

When one tries to investigate a classification with unbalanced data, one has to be very careful in selecting the measure of classification performance. In particular, the *accuracy *alone, is insufficient, because it does not tell us about a performance of predicting the positive class.

The common approaches of deriving alternative evaluation measures are based on *confusion matrix*, where all tested samples are grouped in four categories: "True positives" (TP), "False positives" (FP), "True negatives" (TN) and "False negatives" (FN), with respect to the classification results.

The *sensitivity *and the *specificity *of classifier are defined as follows:

• $sensitivity=\frac{TP}{TP+FN}$

• $specificity=\frac{TN}{TN+FP}$

In order to obtain a single measure of classifier performance, the *sensitivity *and the *specificity *can be integrated into the geometrical mean:

$$G\text{-}mean=\sqrt{sensitivity\times specificity}.$$

### Experimental setup

In this section, we present a comparison of two classification methods – SVM and limJEPClassifier. Both algorithms were tested according to the pipeline and on the data set, described in Methods section. We decided to compare our method with SVM, because it is considered to be the best algorithm used for classification of aCGH data and it has been commonly tested in other works. We did not compare our algorithm with other methods based on JEP's, because traditional strategies of JEP's classification are slow and ineffective.

LimJEPClassifier, as well as the whole experimental pipeline were implemented in the R language [29]. In order to process log2ratio data, we used the freely available "aCGH" package, designed to deal with aCGH data. Another popular R package – "e-1071" was used to perform tests with SVM classifier.

Classifiers were tested with cross-validation where the training to test data ratio was 80%. In order to investigate the influence of the thresholding procedure on the classification results we performed the two runs of experiment with different pairs of positive and negative threshold values: (0.25, -0.25) and (0.5, -0.5). For each pair of threshold levels the cross-validation was repeated 10 times with various numbers of selected features (10, 20,..., 100). Note that feature selection, based on both interval merging and information gain approach, were applied under cross-validation.

### limJEPClassifier vs SVM

**Figure 3:**
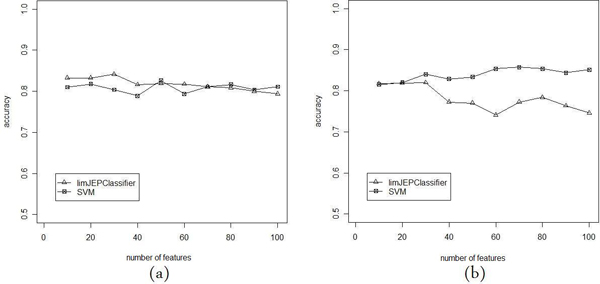
**Accuracy of limJEPClassifier vs SVM**. Comparison of classification accuracy of SVM and limJEPClassifier for two threshold levels: (a) 0.25 and -0.25; (b) 0.5 and -0.5.

**Figure 4:**
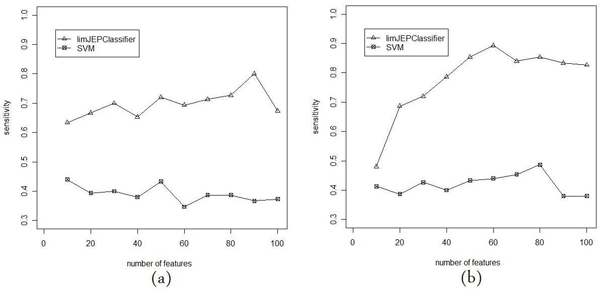
**Sensitivity of limJEPClassifier vs SVM**. Comparison of classification sensitivity of SVM and limJEPClassifier for two threshold levels: (a) 0.25 and -0.25; (b) 0.5 and -0.5.

**Figure 5:**
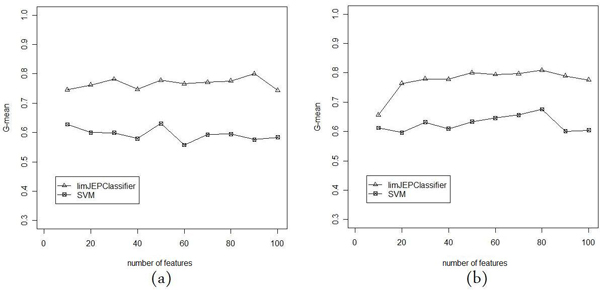
**G-mean of limJEPClassifier vs SVM**. Comparison of G-mean measure of SVM and limJEPClassifier for two threshold levels: (a) 0.25 and -0.25; (b) 0.5 and -0.5.

In Figures 3, 4 and 5 we present the comparison of classification performance of SVM and limJEPClassifier. In each figure the different measure of performance is reported. The (a) and (b) versions of plots correspond to two pairs of threshold levels applied in the experiment.

Figures 3a and 3b show the accuracy of classifiers for various numbers of features. In both cases the values change between 0.74 and 0.85. For the threshold (0.25, -0.25) the differences between SVM and limJEPClassifier are negligible. In figure 3b we see that the accuracy is still similar, however, a little supremacy of SVM could be observed.

On the other hand, it is worth noticing that the number of features have a very low impact on accuracy that is not a proper measure of classification performance in the case of unbalanced data. The last observation seems to confirm this statement.

Figures – 4a and 4b, show the comparison of sensitivity which is the performance of a prediction of the class of affected subjects. We can observe from the results that for both threshold levels, limJEPClassifier is much better than SVM. Furthermore, the sensitivity of SVM is below 0.5 which means that the classifier cannot really distinguish between positive and negative class. The other difference between two classification methods is the way how the sensitivity changes along the X axis. In case of limJEPClassifier the sensitivity is relatively low at the beginning and increases with the number of features, while for SVM we cannot observe such a tendency.

The last pair of plots (Figure 5a and 5b) present the *G-mean *value in which the *sensitivity *and *specificity *are included. Like in the previous case, the limJEPClassifier significantly predominated over SVM.

## Conclusion

It is clear that improving a classification of aCGH data can contribute to great progress in many medical applications. Unfortunately, because of the imperfections of this technology, low signal to noise ratio and rapidly increasing microarray resolutions, classification still remains a very difficult problem.

In this paper, we have investigated the problem of the classification  using DNA copy number data which are characterized by an extremely high dimensionality and unbalanced distribution. We have introduced the concept of limited JEP's and we have shown how they can be applied in aCGH data classification. What is more, we suggest that the performance of a given *limJEP *level can be measured by analysing the structure of the training data.

To confirm the performance of our approach we have developed an experimental pipeline and we have compared limJEPClassifier with SVM. Experiments have been performed using widely tested TP-53 data set [19]. Although the SVM is considered one of the most successful methods of classifying high-throughput data, limJEPClassifier has revealed a much better performance in predicting the class of sick subjects. The main advantage of limJEPClassifier over SVM is that it deals more effectively with unbalanced data. We have not confronted our results with the previous one, presented in article [1], because we claim they are incomparable. We put it, that both experimental setup and results presentation showed in [1] were done in an improper way.

In this research, we have found out that applying limited JEP's can be useful in aCGH data classification. However, this issue should be investigated more deeply in future studies. It would be interesting to test limJEPClassifier with other data sets. Furthermore, it would be valuable to check the influence of different segmentation methods on the performance of limJEPClassifier.

## Competing interests

The authors declare that they have no competing interests.

## Authors' contributions

KW and TG designed all experiments and algorithm of limJEPClassifier. KW directed the study, guided the whole project and assisted the manuscript writing. TG implemented limJEPClassifier and testing pipeline and wrote the manuscript.
